# Targeting Bcl-xL to eliminate chemotherapy-induced tumor dormancy and prevent breast cancer metastasis

**DOI:** 10.1038/s41416-025-03292-y

**Published:** 2025-12-15

**Authors:** Kinjal Gupta, Nicholas Koelsch, Victoria Neely, Laura Graham, Harry D. Bear, Michael O. Idowu, Hisashi Harada, Masoud H. Manjili

**Affiliations:** 1 Department of Microbiology & Immunology, VCU School of Medicine, Richmond, VA USA; 2 Philips Institute for Oral Health Research, VCU School of Dentistry, Richmond, VA USA; 3 VCU Massey Comprehensive Cancer Center, Richmond, VA USA; 4 Department of Surgery, VCU School of Medicine, Richmond, VA USA; 5 Department of Pathology, VCU School of Medicine, Richmond, VA USA

**Keywords:** Apoptosis, Breast cancer

## Abstract

Chemotherapy-induced tumor dormancy is a major barrier to curative cancer therapy, particularly in triple-negative breast cancer (TNBC), where dormant residual cells evade treatment and fuel late relapses. To define survival mechanisms sustaining dormancy, we examined four breast cancer models: HER2-positive murine MMC and human SK-BR-3, and TNBC murine 4T1 and human MDA-MB-231. Dormancy was induced with low-dose FAC (5-Fluorouracil, Adriamycin, Cyclophosphamide). Across all models, dormant cells maintained high Bcl-xL expression. shRNA knockdown of Bcl-xL increased chemotherapy-induced apoptosis and prevented relapse in vitro and in vivo. Pharmacologic inhibition with A-1331852 improved chemotherapy, particularly in TNBC, and transient dosing avoided compensatory Survivin induction. Systemic A-1331852 suppressed relapse but caused off-target toxicity, whereas intratumoral delivery preserved efficacy and safety but failed to eliminate early lung dissemination, as confirmed by ex vivo culture of dormant tumor cells. Notably, disseminated cell frequency inversely correlated with primary tumor size during neoadjuvant chemotherapy, underscoring the need for systemic therapies targeting distant dormant cells. These findings identify Bcl-xL as a central survival factor in chemotherapy-induced dormancy, and suggest that tumor-targeted systemic delivery of A-1331852 may eradicate disseminated dormant cells and prevent metastatic relapse in high-risk TNBC.

## Introduction

Late metastatic relapse remains the principal cause of mortality in breast cancer, often emerging years after apparently curative therapy. A growing body of evidence indicates that chemotherapeutic stress can push a subset of tumor cells into a quiescent, drug-tolerant state known as tumor dormancy [1, 2]. These dormant residual cells evade conventional cytotoxic regimens, retain full malignant potential, and seed lethal recurrences once therapy-induced selective pressure is lifted [3, 4]. Although dormancy has been described across subtypes, its molecular underpinnings—particularly the survival pathways that allow dormant cells to resist apoptosis—remain incompletely defined.

The mitochondrial (cell-intrinsic) apoptotic pathway, governed by the Bcl-2 family, is a key arbiter of cell fate after chemotherapy [5]. Within this family, anti-apoptotic guardians Bcl-2, Mcl-1, and Bcl-xL sequester pro-death BH3-only proteins and directly inhibit the BAX/BAK pore-forming effectors. Bcl-xL is of particular interest in breast cancer: it’s overexpression in triple-negative breast cancer (TNBC) correlates with chemoresistance and promotes metastatic dissemination independent of its canonical anti-apoptotic role [6]. Pharmacologic Bcl-xL antagonists such as A-1331852 have shown potent cytotoxicity in pre-clinical models, yet their development has been hampered by off-target thrombocytopenia and other systemic toxicities [7, 8]. Consequently, the extent to which Bcl-xL sustains chemotherapy-induced dormancy—and whether selective, temporally constrained inhibition can eradicate residual disease without unacceptable toxicity—remains an open question.

Moreover, comparative studies spanning molecular subtypes are scarce. TNBC and HER2-positive tumors differ markedly in intrinsic apoptotic priming, drug sensitivity, and patterns of relapse [9]. It has not been systematically explored whether these subtype-specific traits translate into distinct dormancy survival circuits. Addressing this gap is essential for rational design of combination regimens that pair low-dose, immunogenic chemotherapy - which minimizes collateral damage yet reliably induces immunogenic cell death - with transient Bcl-xL blockade to extinguish residual cells before they can reactivate [10–13].

Here, we investigate the survival circuitry of chemotherapy-induced dormancy across representative TNBC and HER2-positive models. Building on our earlier observation that chemotherapy drives a fraction of MMC cells into dormancy [14], we (i) define the dominant anti-apoptotic dependencies that emerge during this state, (ii) interrogate the functional necessity of Bcl-xL using genetic knockdown and the selective inhibitor A-1331852, and (iii) evaluate clinically relevant delivery strategies to maximize efficacy while mitigating systemic toxicity. By clarifying the role of Bcl-xL in dormant residual disease, our study aims to establish a mechanistic foundation for targeted combination therapies capable of preventing relapse of aggressive breast-cancer subtypes.

## Materials and methods

### Tumor cell lines

The Neu-overexpressing mouse mammary carcinoma (MMC) cell line was derived from spontaneous mammary tumors in FVBN202 mice [15]. MMC cells were seeded at 3 × 10⁶ cells in 75 cm² flasks containing 8 mL RPMI-1640 medium with L-glutamine and 10% fetal bovine serum (FBS). The murine triple-negative breast-cancer (TNBC) line 4T1 was cultured under identical conditions. The human HER2-positive SK-BR-3 line was purchased from ATCC (Manassas, VA) and was seeded at the same density in McCoy’s 5 A medium with L-glutamine and 10% FBS. The human TNBC line MDA-MB-231 was also obtained from ATCC (Manassas, VA) and was maintained at 3 × 10⁶ cells per 75 cm² flask in Dulbecco’s modified Eagle’s medium (DMEM) with L-glutamine and 10% FBS.

### Chemotherapy-induced tumor dormancy model to evaluate the Bcl-xL inhibitor A-1331852

FAC chemotherapy comprised 5 µM 5-fluorouracil (5-Fluorouracil; Sigma-Aldrich, St. Louis, MO), 0.1 µM doxorubicin (Adriamycin; Sigma-Aldrich, St. Louis, MO), and 2 µM cyclophosphamide (Sandoz, Princeton, NJ) for MMC, SK-BR-3, and MDA-MB-231 cells. Because 4T1 cells proliferate more rapidly, they received double these concentrations (10 µM 5-Fluorouracil, 0.2 µM doxorubicin, 4 µM cyclophosphamide). Drugs were first added 4–6 h after seeding—once cells had attached—and the medium was replaced with freshly prepared FAC each day for five consecutive days. The selective Bcl-xL inhibitor A-1331852 (Selleck Chemicals, Houston, TX) was co-administered at 1 µM for MMC, SK-BR-3, and MDA-MB-231, and 2 µM for 4T1, during each FAC exposure and for 24 h after the final (fifth) treatment. Cell viability was not assessed solely by adhesive or suspensive properties. In all cases, viability was additionally confirmed using standard viability dyes during counting to ensure accuracy.

### Bcl-xL knockdown in MMC tumor cells

The Bcl-xL-knockdown MMC line (shBcl-xL MMC) was generated by lentiviral transduction. Lentivirus was produced in HEK-293T cells co-transfected with the shRNA plasmid pLKO.1-shBcl-xL (TRCN0000004685, MilliporeSigma, Burlington, MA), the packaging plasmid psPAX2, and the envelope plasmid pMD2.G (Addgene, Watertown, MA; #12260 and #12259, respectively) using EndoFectin™ Lenti reagent (GeneCopoeia, Rockville, MD) in Opti-MEM® (Gibco ThermoFisher, Waltham, MA) according to the manufacturer’s protocol. MMC cells were seeded at 3 × 10⁵ cells per well in six-well plates and allowed to attach overnight. The next day, medium was replaced and 1 mL of viral supernatant supplemented with 2 µg mL⁻¹ polybrene was added, followed by centrifugation at 2000 rpm for 1 h. After 24 h, the medium was refreshed, and puromycin selection (2 µg mL⁻¹) was initiated 48 h post-infection. Stably transduced cells were expanded and cloned by limiting dilution in RPMI-1640 containing 10% FBS and puromycin. A scrambled shRNA control line (Scr MMC) was generated in parallel using pLKO.1-Scramble (Addgene, Watertown, MA; #1864). HEK-293T producer cells were maintained in DMEM with 10% FBS. All cultures were kept at 37 °C, 5% CO₂ and passaged with 0.25% trypsin-EDTA (Corning Life Sciences, Tewksbury, MA).

### Specifications of SHCLND plasmid TRCN0000004685 (Sigma-Aldrich, St. Louis, MO Cat# SHCLND-NM_009743)

Target Sequence: AGCTGGAGTCAGTTTAGTGAT; Gene Synonym: BCL-XL/S, BCL2L, BCLX, BCLXL, BCLXS, Bcl-X, PPP1R52, bcl-xL, bcl-xS; Cell Type: Hepa 1-6; Method: SYBR; Knockdown: 95%; Version: 1.

### Bcl-xL knockdown media preparation

Puromycin-containing media for maintaining the Bcl-xL knockdown (shBcl-xL) phenotype was prepared by adding puromycin (10 mg/mL stock solution; Fisher Scientific, Waltham, MA; Cat. #40-895-0) to RPMI-1640 medium supplemented with 10% fetal bovine serum to achieve a final concentration of 2 µg/mL. The entire bottle of complete RPMI was supplemented accordingly and used for routine culture of stable shBcl-xL MMC cells.

### Western blot analyses and antibodies

Whole cell lysates were prepared with CHAPS lysis buffer (ThermoFisher scientific, Waltham, MA), 1:100 Protease Inhibitor Cocktail (EDTA-Free, 100X in DMSO) (APExBIO, Houston, TX) was added to the lysis buffer. Protein concentrations were taken using Bradford assay, after which equal amounts of proteins were loaded on SDS-polyacrylamide gels and transferred to nitrocellulose membranes. Next, the membranes were probed with primary antibodies overnight at 4 °C and probed with secondary antibody for 1 h at room temperature before being visualized. Primary antibodies for GAPDH, Bcl-xL, Mcl-1, Bcl-2, Survivin, BIM, Cleaved-Caspase3 and HRP-linked anti-rabbit IgG, HRP-linked anti-mouse IgG for secondary antibody were from cell signaling technology (Beverly, CA). For visualization purposes, the contrast of the entire blot was uniformly adjusted using ChemiDoc software to accommodate overexposed bands.

### Animal models of breast cancer

FVB/N-Tg(MMTVneu)202Mul/J (FVBN202) mice (Jackson Laboratory, Bar Harbor, ME; #002376) were bred in-house to supply animals for studies with the Neu-overexpressing MMC line derived from this strain. MMC cells were harvested with trypsin–EDTA, washed, and resuspended in ice-cold PBS; 2–3 × 10^6^ cells were injected into the fourth mammary fat pad of 10–12-week-old mice. Twenty-four hours later, mice were assigned to low-dose FAC, FAC + A-1331852, or untreated control groups. Scrambled-shRNA MMC (Scr MMC) and Bcl-xL-knockdown MMC (KD MMC) were implanted at 2 × 10^6^ cells, as preliminary experiments showed Scr MMC required fewer cells than parental MMC to achieve comparable tumor take.

BALB/cAnNHsd mice (Inotiv, West Lafayette, IN) were used for 4T1 triple-negative breast-cancer studies. 4T1 cells (5 × 10^4^) prepared as above were injected into the mammary fat pad of 10–12-week-old mice. Forty-eight hours later, animals began low-dose FAC, FAC + A-1331852, or no-treatment protocols. For metastatic assays, tumors were surgically resected under isoflurane anesthesia. Mice were monitored daily for one week and then 2–3 weeks post-surgery. At endpoint, lungs were harvested for ex vivo culture to detect metastatic 4T1 colonies.

These studies have been reviewed and approved by the Institutional Animal Care and Use Committee (IACUC) at Virginia Commonwealth University on animal protocol number AM10167. All methods were performed in accordance with the relevant guidelines and regulations.

### Tumor volume calculation

Tumor volume (mm^3^) is calculated from measures of length (*L*) and width (*W*) of tumors, as measured by a digital caliper according to the below formulation: ((*L* x (*w*^2^))/2) = tumor volume mm^3^.

### In vivo low dose FAC chemotherapy treatment

Both FVBN202 and BALB/c mice received low-dose FAC chemotherapy formulated as 200 µg 5-fluorouracil, 200 µg cyclophosphamide, and 60 µg adriamycin per 20 g mouse (≈10 mg kg⁻¹ 5-Fluorouracil, 10 mg kg⁻¹ CYP, 3 mg kg⁻¹ ADR, adjusted to individual body weight). FVBN202 mice tolerated seven consecutive daily injections, whereas BALB/c mice were less tolerant and therefore received the regimen for four consecutive days.

### In vivo administration of A-1331852

Initially, A-1331852 was administered via oral gavage after solubilizing the 5 mg powder with DMSO and then corn oil at a concentration of 3.16 mM or 25 mg/kg. For administering 1000 µg per 20 g mouse, 240 µL of DMSO was used to solubilize the powder, then 2160 µL of corn oil according to the manufacturers protocol (10% DMSO and 90% corn oil), in order to oral gavage 200 µL per mouse. Due to high-toxicity of even a single dose administration, which has also been seen via intravenous administration [15–17], we adjusted to subcutaneously inject A-1331852 solubilized in PBS with a final concentration of either 3 mM or 4 mM to avoid toxicity and localize the drug near the tumors in mammary fat pads.

### Recovery of metastatic 4T1 from the lungs, ex vivo

For analysis of lung metastases in BALB/c mice, one lung lobe was fixed in 10% neutral-buffered formalin for histological evaluation, while the remaining lung tissue was processed under sterile conditions for ex vivo culture to assess the presence of metastatic 4T1 cells. Briefly, lungs were minced and incubated overnight at 4 °C in 0.25% trypsin-EDTA. The following day, the suspension was incubated at 37 °C for 30 min, then mechanically homogenized to generate a single-cell suspension. Residual tissue fragments were transferred to a 40-µm cell strainer placed in a Petri dish containing 5 mL of PBS and further dissociated using the back of a 5 mL syringe plunger. The resulting suspension was centrifuged at 1200 rpm for 5 min at 4 °C. After discarding the supernatant, cells were treated with 5 mL of ACK lysis buffer on ice for 5 min with intermittent gentle shaking to remove red blood cells. Cells were then washed twice with RPMI + 10% FBS and resuspended in fresh medium for plating in 25 cm² flasks. Representative images were captured on days 2, 4, and 7, and adherent cells were counted on days 4 and 7 to quantify metastatic colony outgrowth.

### Histological analyses

Formalin fixed paraffin embedded (FFPE) tumor and lung tissues were subjected to hematoxylin and eosin (H&E) staining using Dako CoverStainer (Agilent Technologies, Santa Clara, CA). Immunohistochemistry (IHC) was also performed for the proliferation marker Ki67 (Cell Signaling Technology, Danvers, MA; #12202) performed on the Leica BOND Rx, in order to determine the proliferation status of primary tumors and metastatic 4T1 tumor cells in the lungs of Balb/C mice. Histology slides were scanned at 40× magnification using the PhenoImager HT (formerly Vectra Polaris, Akoya Biosciences, Marlborough, MA). Regardless of treatment group, some mice did not show any histologically detectable tumor cells in the lungs, whereas others grew rapidly in which we presented the representative data points.

### Statistical analysis

Two-tailed Student’s *t* tests were applied to the selected end-point measurements for both in vitro and in vivo experiments; the resulting *p* values are shown in the figures for each direct comparison.

## Results

### Chemotherapy-induced tumor dormancy is characterized by predominant Bcl-xL expression

**Fig. 1:**
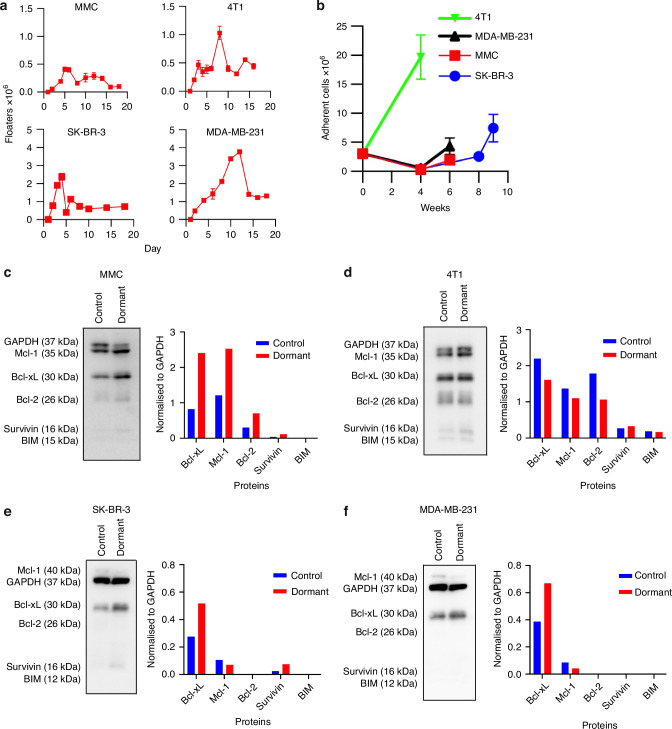
Chemotherapy-induced tumor dormancy is characterized by predominant Bcl-xL expression. **a** Murine or human HER2 positive tumor cells (3 × 10^6^ cells/flask, *n* = 3, MMC or SK-BR-3) or TNBC cells (3 × 10^6^ cells/flask, *n* = 3, 4T1 or MDA-MD-231) were treated with 5 daily doses of FAC (5 µM 5-FU, 2 µM CYP and 0.1 µM ADR, with 4T1 receiving a two-fold higher concentration 10 µM 5-FU, 4 µM CYP, and 0.2 µM ADR), and floating (non-adherent) cells were counted (×10^6^) till day 18, using the trypan blue exclusion method. **b** FAC treated adherent cells were counted (x10^6^) using trypan blue exclusion when relapse regions became apparent (MMC or 4T1, *n* = 3, SK-BR-3 or MDA-MB-231, *n* = 6) *Due to low cell yields, MMC flasks were pooled at each time point; the mean cell number was then calculated by dividing the pooled count by the number of replicates (weeks 3–5, *n* = 3; week 8, *n* = 2). Western blot analyses of MMC (**c**), 4T1 (**d**), SK-BR-3 (**e**) or MDA-MB-231 (**f**) cells collected either before treatment (Control) or during FAC-induced dormancy (Dormant) were performed. Blots were probed for Mcl-1 (Mouse-35 kDa, Human-40 kDa), Bcl-xL (30 kDa), Bcl-2 (26 kDa), Survivin (16 kDa), and BIM (Mouse-15kDa, Human-12kDa). Bar graphs show averaged intensity of protein expression normalized to GAPDH (37 kDa).

We have previously reported that chemotherapy of MMC induces tumor cell death, but some tumor clones enter into the state of dormancy and regrow after the completion of chemotherapy [1–3]. To delineate the survival circuitry sustaining chemotherapy-induced dormancy, we treated Neu-positive murine MMC and HER2+ human SK-BR-3 cells, along with murine 4T1 and human MDA-MB-231 TNBC cells, with low-dose FAC (5-Fluorouracil, Adriamycin, Cyclophosphamide) in vitro. Daily enumeration of non-adherent “floating” cells confirmed ongoing chemotherapy-induced death across all lines (Fig. 1a). Viable adherent cells were then counted, showing that TNBC cultures repopulated more rapidly than their HER2-positive counterparts, indicating a faster relapse trajectory (Fig. 1b). Western blot analysis with the cell lysates before treatment and during dormancy revealed persistent, dominant expression of the anti-apoptotic protein, Bcl-xL in every cell line (Fig. 1c–f). All tumor cell lines showed increased Bcl-xL expression following FAC treatment, except for 4T1 cells, which displayed reduced Bcl-xL levels that nevertheless remained higher than those of Mcl-1 and Bcl-2 (Fig. 1c–f). These findings implicate Bcl-xL as a central survival node in dormant residual disease and provide a strong rationale for combining Bcl-xL inhibition with chemotherapy to eliminate residual tumor cells and prevent relapse.

### shRNA-mediated inhibition of Bcl-xL hinders MMC tumor relapse in vitro and in vivo

**Fig. 2:**
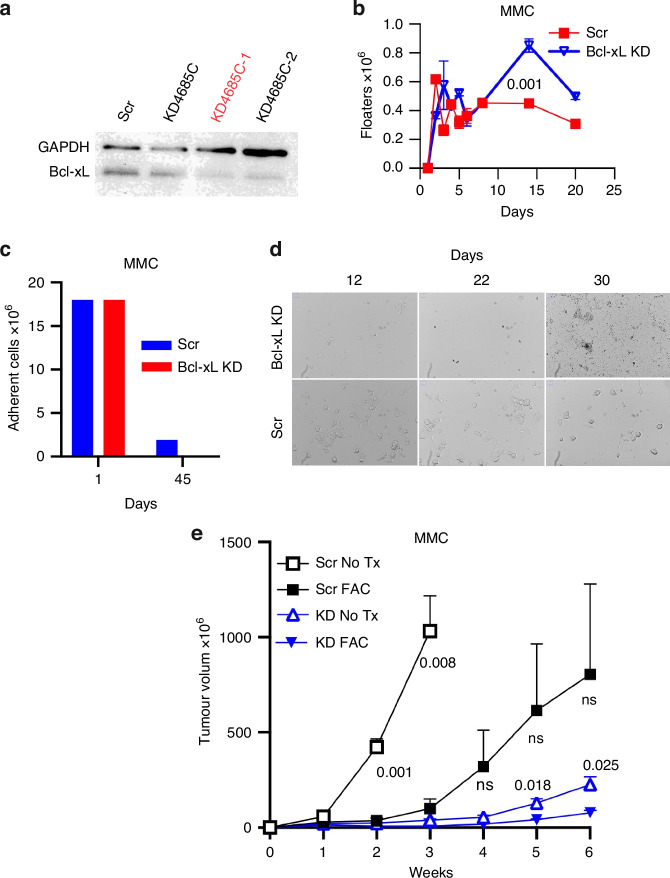
Bcl-xL knockdown hinders MMC tumor relapse. **a** Western blot analysis of scrambled (Scr) control or Bcl-xL knockdown (KD) MMC (KD4685C) cells and its clones (KD 4685C-1,2). **b** Scr MMC and KD MMC (clone 4685C-1) cells (3 × 10^6^ cells/flask, *n *= 3 each) were given a low dose FAC treatment (5 µM 5-FU, 2 µM CYP and 0.1 µM ADR) for 5 days and floating (non-adherent) cells were counted (x10^6^) till day 20, using trypan blue exclusion method. Two-tailed t-test p-values show comparison of Scr and Bcl-xL KD at day 14. **c** Adherent cells were pooled from 6 flasks and were counted (×10^6^) on Day 45, using trypan blue exclusion. **d** Bright-field microscopy images depicting Scr and Bcl-xL KD cells on day 12, 22 and 30 from day 1 of low dose FAC treatment at 10× magnification. **e** Female FVBN202 mice were challenged with 2 × 10^6^ Scr and Bcl-xL KD MMC cells with one group receiving no treatment (Scr No Tx *n* = 3, KD No Tx *n* = 3), others receiving only a low dose FAC treatment (200 µg 5-FU, 200 µg CYP, and 60 µg ADR, per 20 g/mouse, i.p; Scr FAC *n* = 3, KD FAC *n* = 4) for 7 days. Tumor volume (mm^3^) was measured using a digital caliper. Two-tailed t-test p-value shows comparison of KD No Tx vs. KD FAC (0.018 and 0.025) at Weeks 5 and 6 and Scr No Tx vs. Scr FAC (0.001 and 0.008) at Weeks 2 and 3.

In order to determine whether Bcl-xL is the main survival pathway for chemotherapy-induced tumor dormancy in tumor cells, we generated a targeted shRNA-mediated knockdown (KD) of Bcl-xL in MMC cells (KD 4685 C), and established the KD clones with more efficient KD of Bcl-xL (4685C-1,-2) and scramble control MMC cells (Scr MMC) (Fig. 2a). Then we treated Scr MMC and KD MMC (clone 4685C-1) cells with low dose FAC for 5 consecutive days and counted floating dead cells every day, showing that KD MMC cells generated highest levels of dead cells within two weeks (Fig. 2b, *p* = 0.001). By day 45, all KD MMC cells were eliminated, while Scr MMC cells began to regrow (Fig. 2c). Bright-field microscopy images of cells demonstrated that KD MMC cells visibly showed lower number of adherent cells by day 30 compared to Scr MMC control cells, suggesting their sensitivity to apoptosis resulting from Bcl-xL KD (Fig. 2d). By weeks 5 and 6 of culture Bcl-xL KD cells had all died in vitro while the Scr cells persisted (Fig. 2d).

Similar results were obtained in vivo, where FVBN202 mice were inoculated with either Scr MMC or Bcl-xL KD MMC cells. FAC treatment significantly inhibited Scr MMC tumor growth at weeks 2 and 3 (Fig. 2e, *p* = 0.001 and 0.008). Notably, untreated KD MMC tumors exhibited sluggish growth, comparable to FAC-treated Scr MMC (Fig. 2e, *p* = ns). However, when KD MMC tumors were treated with FAC, growth was further suppressed, with significant inhibition observed at weeks 5 and 6 (Fig. 2e, *p* = 0.018 and 0.025). These findings underscore the critical role of Bcl-xL in supporting breast cancer survival and driving relapse.

### Transient blockade of Bcl-xL in conjunction with low dose FAC inhibits relapse of TNBC cell lines and neu positive MMC, but not HER2 + SK-BR-3 in vitro

To investigate the therapeutic efficacy of pharmacological inhibition of Bcl-xL, we used a specific Bcl-xL inhibitor, A-1331852. To optimize the dose, A-1331852 was used either during FAC therapy and one additional day after FAC (FAC/A-1331852) or continued after FAC (FAC/A1331852->) for three weeks. We observed the earliest onset of tumor cell death during FAC chemotherapy when A-1331852 was present (Fig. S1A, Day 4 versus Day 11). Adherent cell counts demonstrated further growth inhibition of FAC-treated dormant cells in the presence of A-1331852 by day 19 (Fig. S1B, FAC: 10^6^ cells vs. FAC/A-1331852; 166,500 and 216,500 cells). Although both A-1331852 dosing protocols demonstrated comparable efficacy, continuous administration following FAC therapy led to the upregulation of the anti-apoptotic protein Survivin, suggesting a compensatory survival mechanism (Fig. S1C). Therefore, we selected the regimen involving A-1331852 administration only during FAC treatment and one day thereafter, to minimize Survivin induction and reduce the risk of escape by dormant tumor cells.

**Fig. 3:**
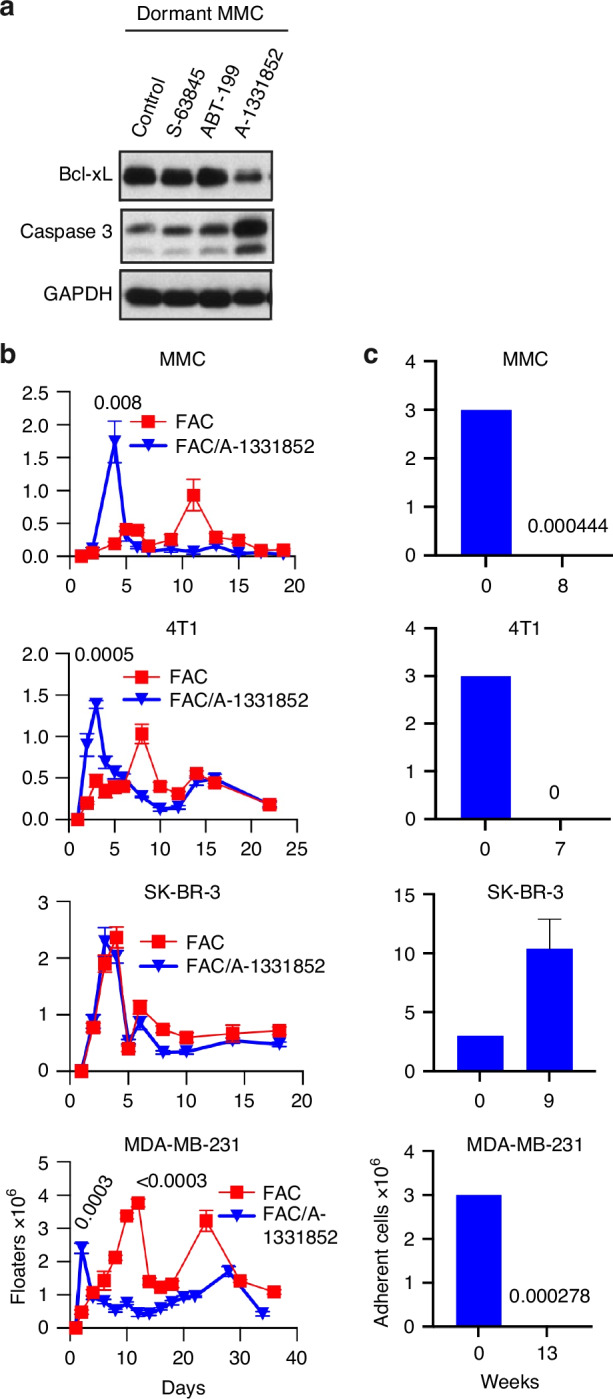
TNBC cells are more responsive than HER2 positive tumor cells to Bcl-xL inhibition during a low dose FAC chemotherapy. **a** Untreated tumor cells (MMC) or FAC-treated dormant cells were treated with 0.1% DMSO (vehicle control) or with the inhibitor of Bcl-2 (ABT-199, 1 µM), Mcl-1 (S-63845, 1 µM) or Bcl-xL (A-1331852, 1 µM) for 24 h. Cell lysates were analyzed for Bcl-xL expression and caspase-3 cleavage by Dr. Harada’s laboratory. Lysates were prepared using CHAPS lysis buffer containing 1 mM dithiothreitol (DTT) in place of 0.2% (w/v) EDTA disodium salt dihydrate, and supplemented with a 1:200 dilution of protease inhibitor cocktail and 1:100 dilutions of phosphatase inhibitor cocktails 2 and 3 (Sigma-Aldrich, St. Louis, MO). **b** MMC, SK-BR-3, and MDA-MB-231 cells (3 × 10^6^ cells/flask, *n* = 3 each) were treated with low-dose FAC chemotherapy or FAC in combination with A-1331852 (5 µM 5-FU, 2 µM CYP, and 0.1 µM ADR for 5 days, along with 1 µM A-1331852 for 6 days). 4T1 cells (3 × 10^6^ cells/flask, *n* = 3) received the same treatments at double the concentration (10 µM 5-FU, 4 µM CYP, and 0.2 µM ADR for 5 days, along with 2 µM A-1331852 for 6 days). Floating (non-adherent) cells were counted (×10^6^) for 20 days for MMC, SK-BR-3, 4T1 cells and up to 40 days for MDA-MB-231 cells, using trypan blue exclusion method. Two-tailed t-test p-values show comparison of FAC/A-1331852 vs. FAC during the first week (Days 2–4 of treatment) for MMC, 4T1, and MDA-MB-231 cells; SK-BR-3 cells at this timepoint was not significant. **c** Adherent cells surviving FAC/A-1331852 treatment were counted (×10^6^) between weeks 7 and 13, using trypan blue exclusion. Due to the low number of surviving cells, replicates for MMC, 4T1, and MDA-MB-231 cells (*n* = 6 each, 3 × 10^6^/ flask) were pooled together to be counted and a mean cell number was then calculated by dividing the pooled count by the number of replicates (i.e., 6); SK-BR-3 cells, which exhibited relapse, were counted individually.

Building on the observation that Bcl-xL expression is sustained during chemotherapy-induced dormancy (Fig. 1c–f), we first assessed the effects of various Bcl-2 family inhibitors on apoptosis of MMC cells, as measured by caspase 3 cleavage. Among these, the selective Bcl-xL inhibitor, A-1331852 induced the highest levels of caspase 3 activation in chemotherapy-induced dormant tumor cells (Fig. 3a). We then evaluated the efficacy of A-1331852 across multiple breast cancer subtypes. All tumor cell lines except SK-BR-3 exhibited significant increase of apoptosis during FAC therapy in the presence of A-1331852 (Fig. 3b). Evaluation of viable adherent cells revealed distinct dormancy dynamics across breast cancer subtypes. In HER2⁺ models, murine MMC cells exhibited prolonged dormancy lasting up to 8 weeks, while human SK-BR-3 cells relapsed after low-dose FAC treatment, with recurrence evident by 9 weeks (Fig. 3c). In contrast, TNBC cell lines displayed heightened sensitivity to A-1331852 following dormancy induction. In the 4T1 model, A-1331852 treatment eliminated all viable cells by 7 weeks post-FAC, whereas MDA-MB-231 cells maintained a stable, non-relapsing dormant state for up to 13 weeks (Fig. 3c, Fig. S2). However, the greater efficacy of A-1331852 in TNBC compared to MMC was not associated with higher levels of caspase 3 cleavage (Fig. S3). These findings underscore Bcl-xL as a critical survival factor for dormant TNBC cells and demonstrate that pharmacologic inhibition with A-1331852 can prevent recurrence. This highlights its potential as a dormancy-targeted adjuvant strategy for triple-negative breast cancer.

### A-1331852-mediated Bcl-xL inhibition enhances low-dose FAC chemotherapy to prevent tumor relapse in FVBN202 mice, but causes systemic off-target toxicity

**Fig. 4:**
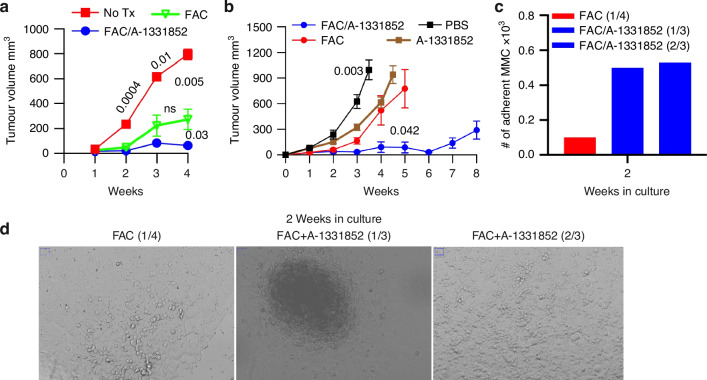
Intratumoral administration of A-1331852 minimizes off-target toxicity while preserving its anti-tumor efficacy. **a** Female FVBN202 mice were challenged with 3 × 10^6^ MMC cells in the mammary pad region, and on the following day, they were given by intraperitoneal injections with 9 daily doses of FAC alone (FAC) (200 µg 5-Fu, 200 µg CYP, and 60 µg ADR per 20 g/mouse, *n* = 3) or combined with oral gavage of a Bcl-xL inhibitor A-1331852 (FAC/A-1331852, *n* = 6) on days 13 and 20. Control group remained untreated (No Tx, *n* = 3). Two-tailed t-test *p*-value shows comparison of No Tx vs. FAC (0.0004, 0.01 and 0.005) on week 2, 3, 4 and a comparison of FAC vs. FAC/A-1331852 (0.03) on week 4. **b** Female FVBN202 mice were challenged with 3 × 10^6^ MMC cells. On the following day, mice received 7 daily doses of FAC (200 µg 5-Fu, 200 µg CYP, and 60 µg ADR per 20 g/mouse, *n* = 4, i.p) or FAC combined with intratumor injection of A-1331852 (98.8 µg/mouse) on days 5, 6, 7, 9, 14, 18, 28, and 42 (*n* = 4; Day 42 only *n* = 2). Control groups were treated with intratumoral injection of A-1331852 on days 5, 6, 7, 9, 14, 18, and 28 (*n* = 4), or PBS on days 5, 6, 7, 9, 14, 18, (*n* = 3). Two-tailed t-test *p* value shows comparison of FAC vs. FAC/A-1331852 (0.042) at week 5, and PBS vs. A-1331852 on day 24 (0.003). **c** Female FVBN202 mice were challenged with 3 × 10^6^ MMC cells and the next day received 7 daily doses of FAC (200 µg 5-Fu, 200 µg CYP, and 60 µg ADR per 20 g/mouse, *n* = 4, i.p) or FAC combined with intratumor injection of A-1331852 (98.8 µg/mouse) on days 5, 6, 7, and 9 (*n* = 3). Lungs were collected at endpoint and cultured ex vivo; showing tumor growth in 1 out of 4 FAC and 2 out of 3 FAC/A-1331852 groups, 2 weeks after ex vivo culture. **d** Representative brightfield microscopy images of FAC (1 of 4) or FAC/A-1331852 (1 and 2 of 3, 1 of 3 cells grew densely and localized while 2 of 3 grew more evenly), 2 weeks after ex vivo lung culture captured at 10× magnification, showing regrowth of MMC cells.

Because of the known off-target toxicity associated with intravenous administration of A-1331852 [16–18], we opted for systemic delivery via oral gavage. FVBN202 mice were inoculated with 3 × 10⁶ MMC cells in the mammary fat pad and assigned to receive either no treatment, low-dose FAC chemotherapy for 9 consecutive days, or FAC in combination with oral A-1331852 administered on days 13 and 20. While FAC alone induced a transient dormancy phase lasting 2–3 weeks, tumor regrowth was observed by week 4 (Fig. 4a). In contrast, co-administration of A-1331852 during FAC treatment prevented relapse during the same period; however, animals became moribund, consistent with systemic toxicity associated with A-1331852 by week 4 (Fig. 4a).

To circumvent this limitation, a separate cohort received intratumoral injections of A-1331852 during FAC therapy. Control mice received intratumoral PBS injections to account for mechanical disruption. Notably, A-1331852-treated mice exhibited a similar pattern of tumor growth compared to that of low-dose FAC alone, yet demonstrating a significant tumor inhibition compared to PBS control group (Fig. 4b, *p *= 0.003). The FAC/A-1331852 group showed significant tumor inhibition compared to FAC or A-1331852 alone by week 5 (*p* = 0.042) as well as prolonged survival from 5 to 8 weeks (Fig. 4b). Ex vivo evaluation of lungs demonstrated that local inhibition of tumor growth at the primary site of the FAC/A-1331852 group was associated with higher rates on metastasis dormancy compared to the FAC-treated group (Fig. 4c, d). Importantly, reducing the MMC cells inoculum from 3 × 10⁶ (Fig. 4a) to a suboptimal 2 × 10⁶ inoculum resulted in slower tumor proliferation in the PBS control group and a diminished response to FAC (Fig. S4). Under this condition, A-1331852 demonstrated enhanced efficacy (Fig. S4), suggesting that Bcl-xL inhibition may sensitize tumors with low proliferative indices that are otherwise less responsive to chemotherapy.

### Intratumoral administration of A-1331852 during low-dose neoadjuvant FAC chemotherapy inhibits primary tumor growth, but fails to suppress distant metastasis in the 4T1 tumor model

**Fig. 5:**
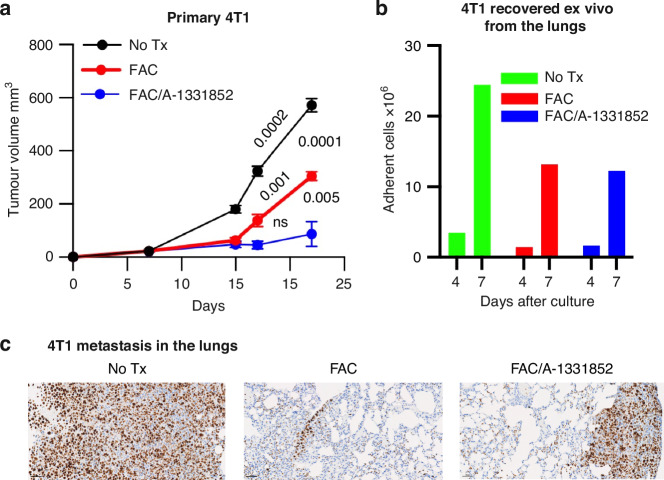
Intratumor inhibition of Bcl-xL in combination with FAC chemotherapy stops 4T1 tumor relapse in vivo. **a** Female Balb/C mice were challenged with 50,000 4T1 cells in the mammary fat pad region, and after 48 h given no treatment (No Tx; *n* = 4), four daily doses of FAC (FAC; 200 µg 5-Fu, 200 µg CYP, and 60 µg ADR per 20 g/mouse, *n* = 4, i.p) or FAC along with intratumor injections of A-1331852 (131.8 µg/ mouse on day 3 and 4) (FAC/A133; *n* = 4). Tumor volume was measured using a digital caliper. Two-tailed t-test *p* values show comparison of No Tx vs. FAC (0.0001) and FAC vs. FAC/A-1331852 (0.005) on Day 22; also, comparisons of Day 17 vs. 22 for No Tx (0.0002), FAC (0.001), FAC/A-1331852 (ns). **b**, **c** Three weeks after 4T1 tumor challenge and completion of the neoadjuvant therapies, tumors were removed, and animals were sacrificed 2- or 3-week post-surgery. Lungs were collected from each group and were cultured ex vivo to recover metastatic tumor cells. The bar graph shows representative adherent cell counts (×10^6^) from each group (**b**) or subjected to IHC staining with Ki-67, showing representative 20× images of highly proliferating (dark brown), indolent tumor cells (light brown) or quiescent dormant cells (blue color) (**c**).

To determine whether administration of A-1331852 during neoadjuvant FAC chemotherapy could impact both primary and metastatic tumors in the absence of adjuvant treatment, we utilized the 4T1 tumor model, which naturally metastasizes to the lungs following surgical resection of the primary tumor. Mice treated with low-dose FAC alone exhibited initial tumor suppression compared to the untreated group (*p* = 0.0001), but tumor regrowth was observed by week 3 (Fig. 5a, *p* = 0.001). In contrast, combinatorial treatment with A-1331852 significantly prolonged tumor dormancy by effectively inhibiting primary 4T1 mammary tumor growth (Fig. 5a, *p* = 0.005) and prolonged tumor dormancy, with no evidence of tumor regrowth during the observation period (Fig. 5a, *p* = ns). Following surgical resection of 4T1 tumors, animals were monitored and sacrificed when passed 20% weight loss. Ex vivo culture of lung tissue revealed recovery of metastatic 4T1 cells in all groups (Fig. 5b). Immunohistochemical analysis of lung sections demonstrated a higher frequency of Ki-67^high^ proliferating tumor cells in untreated animals compared to treated groups (Fig. 5c). These findings suggest that local administration of A-1331852 effectively suppresses primary tumor growth but does not reach distant sites, such as the lungs, sufficiently to inhibit metastatic outgrowth. The A-1331852 group also showed significant tumor inhibition compared to PBS control group (Fig. S5A). Such inhibition of primary 4T1 tumors was associated with higher rates of lung metastasis reflected in higher rates of tumor relapse from ex vivo lung cultures (Fig. S5B, C).

## Discussion

Our findings provide compelling evidence that Bcl-xL is a critical survival determinant in chemotherapy-induced tumor dormancy, particularly in TNBC. Across both in vitro and in vivo systems, we demonstrate that low-dose FAC chemotherapy induces tumor cell death while permitting a subset of residual cells to enter dormancy—a clinically significant state associated with eventual relapse [14, 19, 20]. Notably, we observed persistent, dominant expression of the anti-apoptotic protein Bcl-xL in dormant cells across multiple tumor models, including murine MMC, human SK-BR-3 (HER2 + ), murine 4T1, and human MDA-MB-231 (TNBC) cells, reinforcing the unique role of Bcl-xL in maintaining cell survival during dormancy.

Using both genetic and pharmacologic approaches, we demonstrated that loss or inhibition of Bcl-xL sensitizes dormant tumor cells to apoptosis, effectively inhibiting tumor relapse in vitro and in vivo. Notably, shRNA-mediated knockdown of Bcl-xL resulted in complete elimination of MMC-derived dormant cells, confirming Bcl-xL’s functional necessity. Consistent with these findings, pharmacological blockade using A-1331852 during low-dose chemotherapy induced robust apoptotic responses in TNBC and Neu-positive murine cell lines but not in HER2-positive SK-BR-3 cells, suggesting subtype-specific survival dependencies. To this end, Hsp70 has been shown to regulate apoptosis in SK-BR-3 cells independently of Bcl-xL and Bcl-2 [21], whereas TNBC cells rely primarily on Bcl-xL for survival during chemotherapy [22–25]. These results align with prior literature describing Bcl-xL overexpression in aggressive breast cancers and its association with therapeutic resistance [26, 27].

Our data also highlight an important therapeutic window: transient inhibition of Bcl-xL during and immediately following chemotherapy was sufficient to eliminate dormant cells while avoiding compensatory upregulation of Survivin, an alternative anti-apoptotic protein. This temporally restricted strategy builds on emerging evidence that prolonged BH3-mimetic therapy may induce resistance mechanisms [28, 29], reinforcing the need to tailor the duration of Bcl-xL inhibition to phases of maximal tumor cell vulnerability.

A major challenge in clinical translation of Bcl-xL inhibitors is their systemic toxicity, including thrombocytopenia and cardiovascular effects [30, 31]. Indeed, systemic administration of A-1331852 in our murine tumor model suppressed tumor relapse but resulted in severe toxicity. In contrast, intratumoral delivery maintained therapeutic efficacy while avoiding systemic effects, highlighting the importance of drug localization. However, this approach was insufficient to inhibit metastatic outgrowth in the 4T1 model, likely because of limited drug bioavailability at distant sites - a limitation consistent with earlier reports on local delivery strategies. Importantly, our lung ex vivo culture experiments confirmed that tumor cell dissemination occurs very early during primary breast cancer growth, in line with clinical evidence of early micrometastasis in patients [32, 33]. We found that disseminated dormant tumor cells could be recovered from lungs even during neoadjuvant chemotherapy, and their frequency was inversely correlated with primary tumor size, suggesting that systemic spread precedes overt tumor progression. These observations reinforce that systemic treatments targeting already disseminated dormant tumor cells are essential to prevent metastatic recurrence. Among such treatments, tumor-targeted systemic delivery of A-1331852 represents a promising strategy to eliminate residual disease, and combining this with dormancy-specific immunotherapy [34] could provide durable immune surveillance against disseminated clones.

These findings suggest that while Bcl-xL inhibition is a promising approach to eradicate residual dormant cells and prevent relapse, its clinical success hinges on the development of tumor-targeted systemic delivery systems. Strategies such as PEGylated nanoparticles, antibody-drug conjugates, or peptide-directed carriers may enable systemic distribution while restricting activity to tumor tissues, thereby overcoming the toxicity barrier. Such platforms are currently under investigation and could be integrated into neoadjuvant or adjuvant therapy regimens for high-risk breast cancer patients.

## Conclusions

This study provides compelling preclinical evidence that Bcl-xL is a key mediator of chemotherapy-induced dormancy and relapse in TNBC. Transient inhibition of Bcl-xL during chemotherapy enhances tumor clearance, eliminates residual disease, and delays recurrence - provided toxicity is managed. Future efforts should prioritize the development of tumor-targeted Bcl-xL inhibitor delivery platforms to achieve safe and effective systemic treatment. In parallel, a personalized vaccine strategy targeting dormancy-associated antigens could be leveraged to generate durable immune surveillance against residual dormant clones [14, 35, 36], thereby preventing their reactivation and distant recurrence following completion of chemotherapy combined with A-1331852. Together, these approaches provide a transformative framework for eradicating minimal residual disease, tackling metastatic relapse - the leading cause of mortality in aggressive breast cancer subtypes - and advancing the field toward curative therapy.

### Ethics approval and consent to participate

All original animal procedures were approved by the Institutional Animal Care and Use Committee (IACUC) of at Virginia Commonwealth University on animal protocol number AM10167, and conducted in accordance with relevant guidelines and regulations.

## Supplementary information

Supplementary files

## Data Availability

The datasets generated and/or analyzed during the current study are available from the corresponding author on reasonable request.
