# Supplementary material for: Targeting Bcl-xL to eliminate chemotherapy-induced tumor dormancy and prevent breast cancer metastasis
Source: Br J Cancer. 2025 Dec 15;134(4):676–84. doi: 10.1038/s41416-025-03292-y (PMC12859104; doi:10.1038/s41416-025-03292-y)
Supplement: Supplementary file 1 — Supplementary files [file 41416_2025_3292_MOESM1_ESM.pdf]

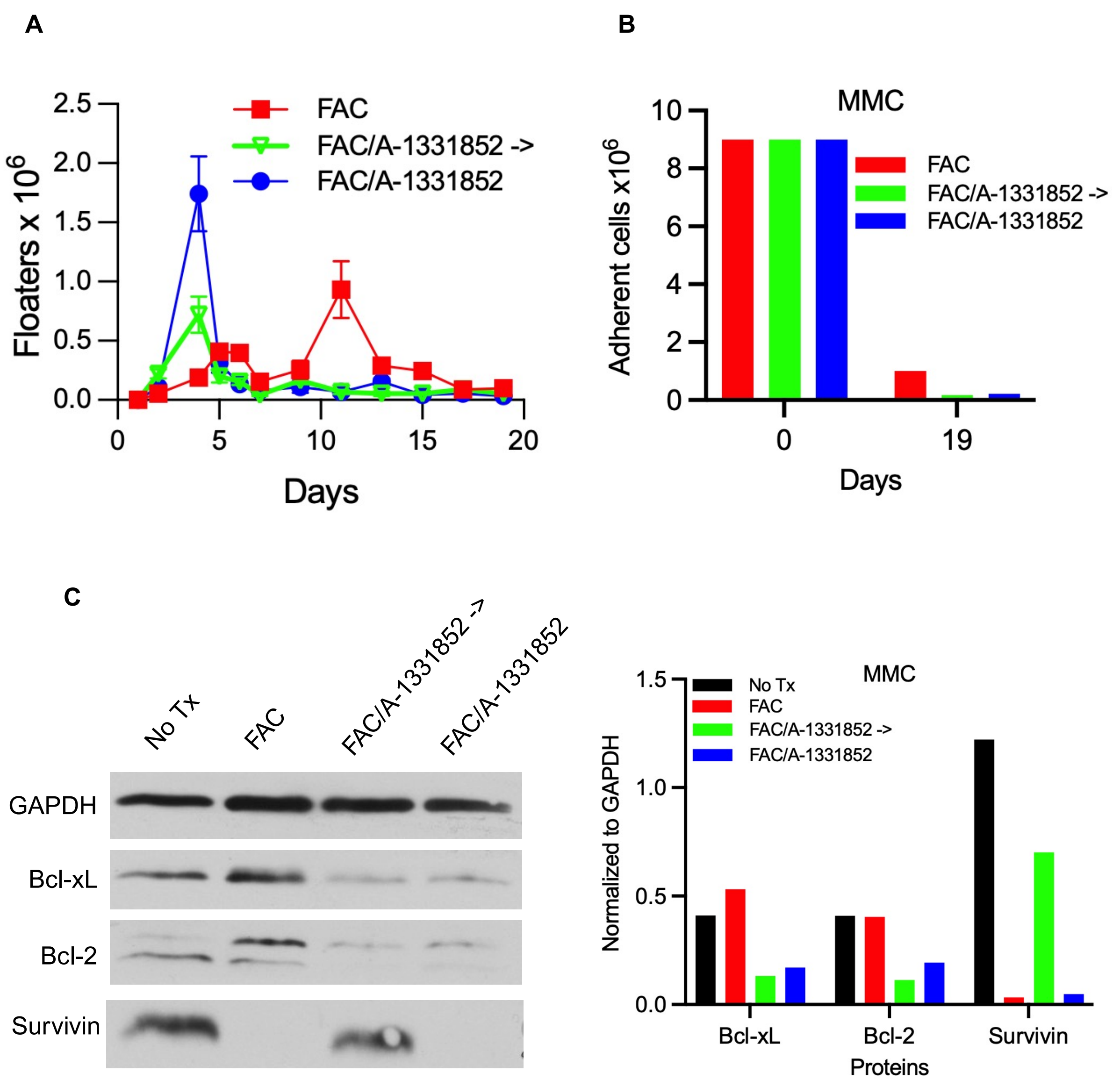

Figure S1. Transient inhibition of Bcl-xL prevents MMC tumor relapse. A) MMC ($3x10^6$/flask) were given a low dose FAC treatment (FAC: 5 µM 5-FU, 2 µM CYP and 0.1 µM ADR, for 5 days; n = 3) and Bcl-xL inhibitor, A-1331852 dosage was optimized by administering 1 µM on every media change during and after low dose FAC (FAC/A-1331852→, n = 3) or for 6 days with low dose FAC treatment (FAC/A-1331852, n = 3). Floating (non-adherent) cells were counted ($x10^6$) for 19 days, using trypan blue exclusion method. Two-tailed t-test p-values show comparison of FAC/A-1331852→ vs. FAC and FAC/A-1331852 vs. FAC on days 4 and 11. **B)** MMC adherent cells were pooled from all flasks (n = 3 per group) and counted ($x10^6$) during dormancy on day 19, using trypan blue exclusion. **C)** Western blot analysis of MMC without treatment (No Tx), and dormant MMC post low dose FAC (FAC), FAC/A-1331852→, and FAC/A-1331852 treatment. Bar graph shows average intensity of protein expression of Bcl-xL (30kDa), Bcl-2 (26kDa) and Survivin (16kDa) normalized to GAPDH (37kDa).

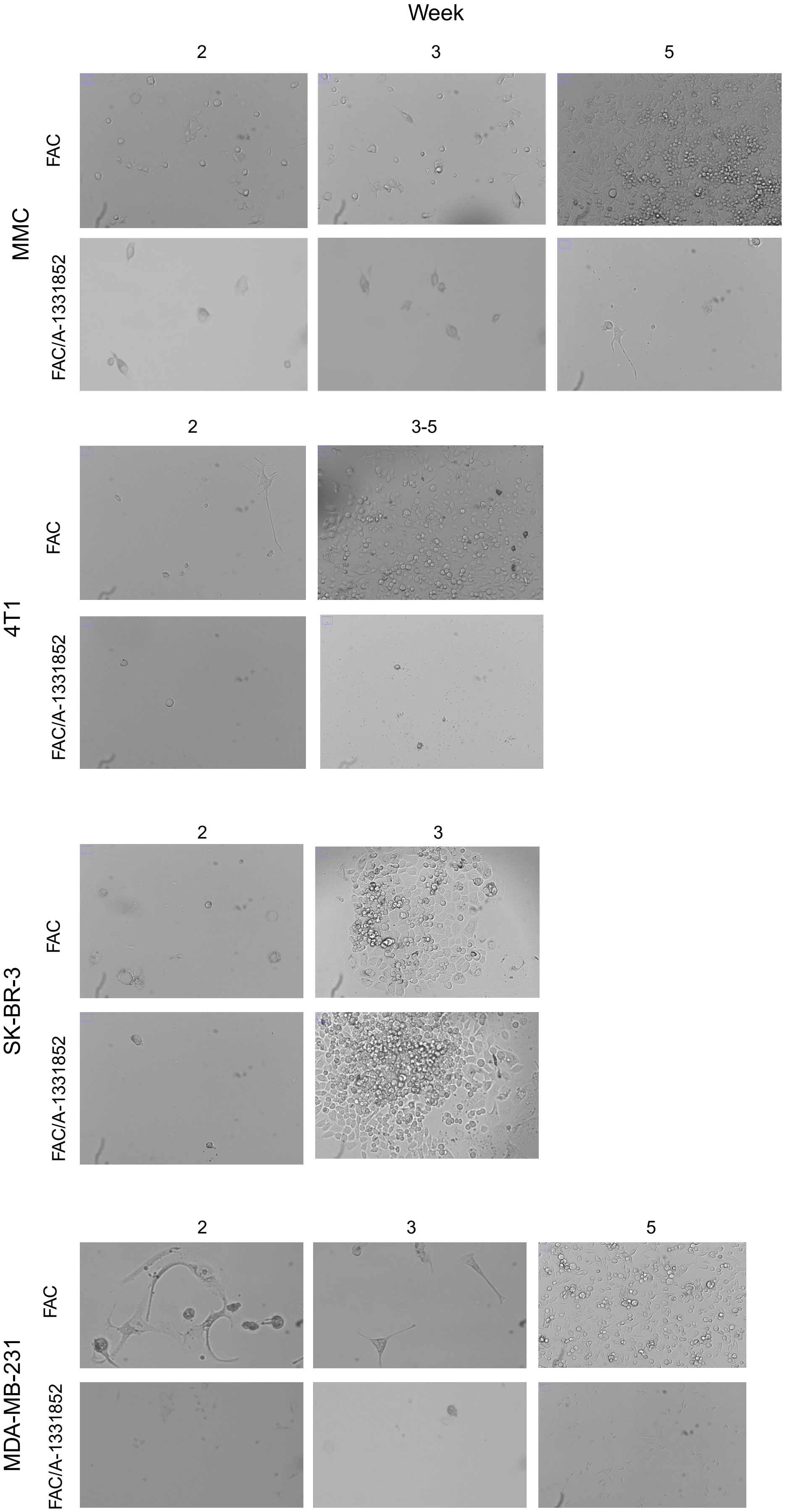

Figure S2. Inhibition of Bcl-xL in combination with chemotherapy impairs survival of TNBC and Neu-expressing MMC cells. Bright-field microscopy images of MMC (upper panel), 4T1 (upper middle panel), SK-BR-3 (lower middle panel), and MDA-MB-231 (lower panel) cells treated with FAC or FAC/A-1331852 from weeks 2 to 5, captured at 10× magnification.

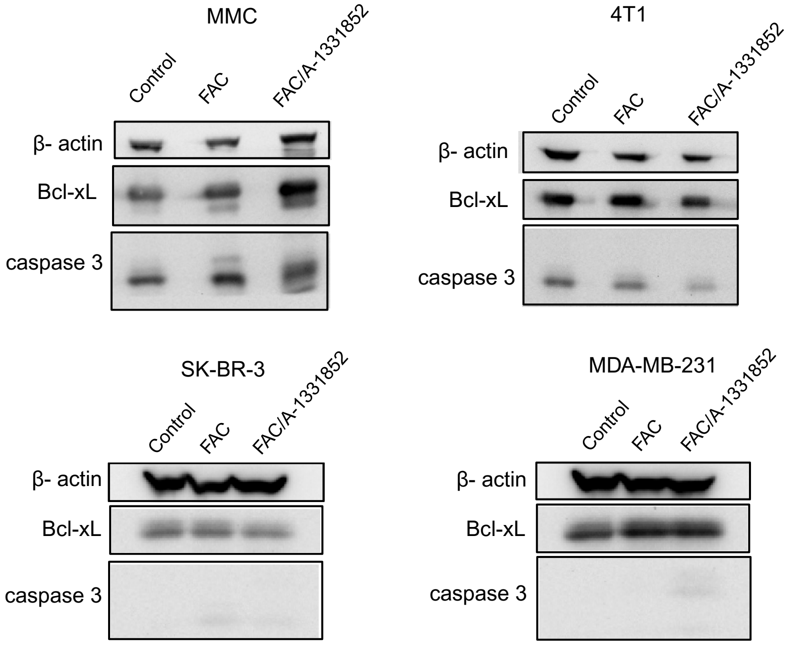

Figure S3. Caspase 3 cleavage following the inhibition of Bcl-xL in combination with chemotherapy. Western blot analysis of MMC (upper left panel), 4T1 (upper right panel), SK-BR-3 (lower left panel), and MDA-MB-231 (lower right panel) cells was performed. Lysates were made from untreated (Control) as well as cells treated with Low-dose FAC for three days either with or without A-1331852. The blots were probed for Bcl-xL and Cleaved Caspase 3.

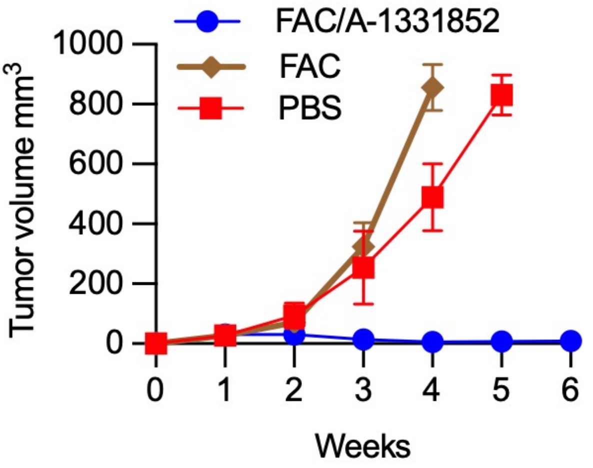

Figure S4. Intratumoral administration of A-1331852 minimizes off-target toxicity while preserving its anti-tumor efficacy. Female FVBN202 mice were challenged with 2x10^6 MMC cells. On the following day, mice received seven daily doses of FAC (200 µg 5-Fu, 200 µg CYP, and 60 µg ADR per 20 gram/mouse, n=3, i.p) or FAC combined with intratumor injection of A-1331852 (98.8 µg/ mouse) on days 5, 6, 7 and 9, (n=9). Control group was treated with intratumoral injection of PBS on days 5, 6, 7 and 9, (n=3).

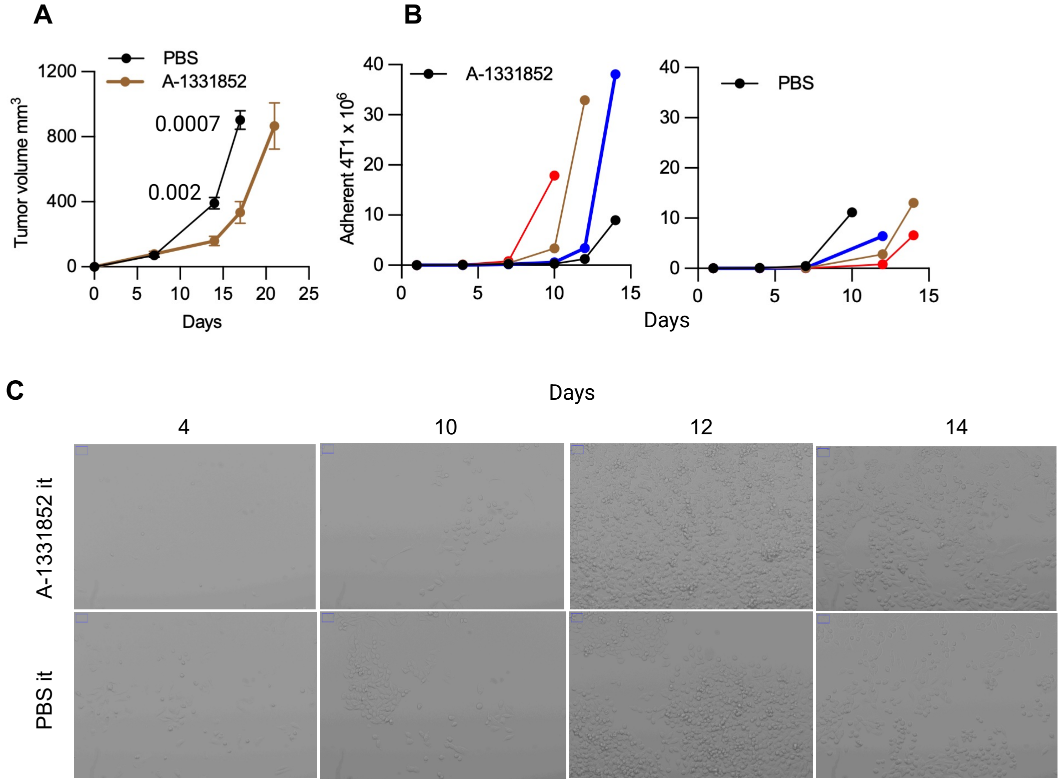

Figure S5. Intratumoral administration of A-1331852 alone fails to inhibit 4T1 relapse and lung metastasis. A) Female Balb/C mice were challenged with 50,000 4T1 cells in the mammary pad region and given A-1331852 (131.75 µg/ mouse on day 3 and 4; A-1331852; n=4) or PBS (day 3 and 4; PBS it; n=4). Two-tailed t-test p-value shows comparison of PBS and A-1331852 groups (0.002 and 0.0007) on days 14 and 17, respectively. **B)** Once tumors reached humane endpoint, lungs were collected from each group and were cultured, *ex vivo*, to recover metastatic tumor cells. Line graphs show regrowth in days from each mouse given PBS (n=4) or A-1331852 (n=4); individual lines represent replicate regrowth dynamics. **C)** Representative bright-field microscopy images of 4T1 *ex vivo* lung cultures treated with A-1331852 it (upper panel) or untreated PBS control (lower panel) on day 4, 10, 12, 14.
